# Aggressive Plattenepithelkarzinome auf dem Boden eines Lichen ruber mucosae: Bericht über drei Fälle

**DOI:** 10.1111/ddg.70174x

**Published:** 2026-08-04

**Authors:** Neda Cramer, Dagmar Wilsmann‐Theis, Michael P. Schön, Jörg Wenzel, Julia Gallwas, Rotraut Mössner

**Affiliations:** ^1^ Klinik für Dermatologie Venerologie und Allergologie Universitätsmedizin Göttingen, Göttingen, Deutschland; ^2^ Zentrum für Hautkrankheiten Abteilung für Dermatologie und Allergologie Universitätsklinikum Bonn Bonn Deutschland; ^3^ Abteilung für Gynäkologie und Geburtshilfe Universitätsklinikum Göttingen Göttingen Deutschland

**Keywords:** Malignität, metastasiertes Plattenepithelkarzinom, Mukosaler Lichen planus, oraler Lichen planus, Vulva‐Lichen planus, Malignancy, Metastatic Squamous Cell Carcinoma, Mucosal Lichen Planus, Oral Lichen Planus, Vulvar Lichen Planus

Sehr geehrter Herausgeber,

Der Lichen planus (LP) ist eine chronisch‐entzündliche Hautkrankheit, die auch die Schleimhäute betreffen kann, wobei am häufigsten die Mundschleimhaut und die Genitalschleimhaut betroffen sind.^1^, ^2^ Der Lichen ruber mucosae (LRM) gilt als Präkanzerose; maligne Transformationen werden bei 0,8 % bis 10 % der Patienten mit oralem LP (OLP) sowie bei bis zu 2,5 % der Betroffenen mit vulvärem LP (VLP) beschrieben,^3^, ^4^, ^5^ und aus LP hervorgehende Plattenepithelkarzinome (SCC) sind als Komplikation bekannt.

In dieser Fallserie berichten wir über drei Patientinnen mit metastasiertem SCC auf dem Boden eines LRM, bei denen rasche Krankheitsverläufe jeweils letal ausgingen (Tabelle 1). Ziel ist es, auf die Gefahr fulminanter, hochdynamischer und metastasierender Verläufe bei LRM aufmerksam zu machen.

Patientin 1 ist eine 63‐jährige Frau mit LRM, der sich vor 4,5 Jahren genital manifestierte und fünf Monate später auch oral auftrat. Eine differenzierte vulväre intraepitheliale Neoplasie (dVIN) wurde erstmals 1,5 Jahre nach Krankheitsbeginn diagnostiziert und von der niedergelassenen Gynäkologin mittels Laservaporisation behandelt. Anschließend erfolgte über sechs Monate eine immunsuppressive Therapie des LRM mit intravenöser Dexamethason‐Pulstherapie und Methotrexat, die vor einer geplanten Cholezystektomie beendet wurde. Nach weiteren 1,5 Jahren wurde erneut eine dVIN festgestellt und behandelt. Drei Monate später wurde ein schlecht differenziertes, nicht HPV‐assoziiertes SCC der Vulva diagnostiziert. Die Ausbreitungsdiagnostik zeigte suspekte inguinale sowie links pelvine Lymphknoten und eine Infiltration der Urethra. Nach operativer Therapie wurde eine Radiochemotherapie mit Cisplatin begonnen. 2,5 Monate später kam es zu einer fulminanten Progression mit kutanen Metastasen (Lymphangiosis carcinomatosa, Abbildung 1a,b), einer Peritonealkarzinose sowie axillären Lymphknotenmetastasen. Die Patientin verstarb drei Wochen später.

**ABBILDUNG 1:**
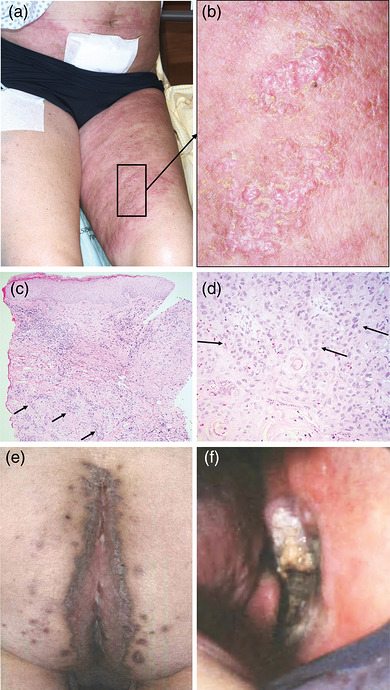
Klinische und histologische Abbildungen der Patientinnen 1, 2 und 3. Patientin 1 mit metastasiertem Vulvakarzinom 2,5 Monate nach Operation und Radiochemotherapie mit Peritonealkarzinose, axillären Lymphknotenmetastasen und Lymphangiosis carcinomatosa (a); letztere in einer Detailaufnahme des linken ventromedialen Oberschenkels (b). Histologische Abbildungen von Patientin 2: Unter einem keratinisierten Plattenepithel ohne Atypien findet sich ein infiltrierendes, mäßig differenziertes Plattenepithelkarzinom (schwarze Pfeile), das das gesamte Präparat einnimmt. Die histologischen Aufnahmen zeigen das Karzinom in 5‐facher (c) und 20‐facher Vergrößerung (d). Patientin 3 mit ausgeprägtem anogenitalem Lichen planus (e) zum Zeitpunkt der Diagnose eines Oropharynxkarzinoms am linken dorsalen Alveolarkamm (f).

Patientin 2 ist eine 64‐jährige Frau mit seit drei Jahren bestehendem VLP. Zwei Jahre nach Diagnosestellung entwickelte sich unter Therapie mit Acitretin ein vulväres Karzinom auf dem Boden des VLP (Abbildung 1c,d). In der CT‐Untersuchung zeigte sich eine Lymphknotenmetastase in der rechten Leiste sowie beidseits suspekte Lymphknoten im parailiakalen Bereich. Es erfolgte eine beidseitige laparoskopische inguinale Lymphadenektomie mit anschließender Chemotherapie mit Cisplatin und Paclitaxel, unter der es jedoch zu einem Progress kam. Nach anschließender Vulvektomie mit beidseitiger inguinaler Lymphadenektomie wurden das Tumorbett und die Leistenregion bestrahlt. Etwa fünf Monate später trat eine ausgedehnte Peritonealkarzinose auf und die Patientin verstarb.

Patientin 3 ist eine 77‐jährige Frau mit seit 33 Jahren bestehendem OLP (Abbildung 1e). 32 Jahre nach Diagnosestellung entwickelte sich auf Bodem des OLP ein Oropharynxkarzinom (Abbildung 1f). Es erfolgten eine Laserresektion, eine *Neck Dissection* sowie die Exstirpation der linken submandibulären Lymphknoten, wobei histologisch ein mäßig differenziertes SCC mit Kapseldurchbruch bestätigt wurde. Eine adjuvante Radiochemotherapie mit Cisplatin wurde begonnen, jedoch nach vier Wochen aufgrund ausgeprägter Übelkeit und Diarrhö sowie einer Mukositis Grad III–IV abgebrochen. 13 Monate später zeigten die Histologie und die CT‐Untersuchung ein ausgedehntes, nicht resektables lokales Rezidiv. Aufgrund der vorausgegangenen Strahlenexposition wurde auf eine erneute Radiotherapie verzichtet. Es wurde eine palliative Tracheotomie mit mukokutaner Anastomose durchgeführt. Die Patientin verstarb 15 Wochen später.

Das Potenzial der malignen Entartung auf dem Boden eines LRM wird seit den 1990er‐Jahren zunehmend in Fallberichten diskutiert.^6^, ^7^, ^8^ Ob sich der LRM direkt in ein SCC transformiert oder ob die chronische Entzündung des LRM die Karzinogenese eines unabhängig vom LRM mutierten Tumors begünstigt, ist bislang nicht abschließend geklärt. Die Literatur berichtet, dass beide Mechanismen möglich sind und auch koexistieren können.^9^, ^10^, ^11^, ^12^, ^13^ Exom‐Sequenzierungsanalysen zeigen, dass Fälle eines OLP, die in ein orales SCC übergehen, wiederkehrende Mutationen in Genen wie TP53, CELSR1, CASP8 und KMT2D aufweisen, während diese Veränderungen in nicht transformierenden OLP‐Läsionen nicht nachweisbar sind.^14^ Da molekulargenetische Untersuchungen auf diese Mutationen bislang nicht klinisch etabliert und für die Früherkennung validiert sind, stellen die engmaschige klinische Überwachung und die histopathologische Diagnostik weiterhin den Therapiestandard dar.^15^

Chronisch‐entzündliche Läsionen der Vulva, wie der LRM und der Lichen sclerosus et atrophicus, können zur Entwicklung einer dVIN führen, die im Vergleich zu anderen, HPV‐assoziierten vulvären Präkanzerosen ein höheres Progressionsrisiko zum invasiven SCC aufweist und zudem innerhalb von drei bis zwölf Monaten deutlich schneller fortschreitet.^16^, ^17^ Als Therapie der Wahl für dVIN wird in den deutschen und europäischen Leitlinien die chirurgische Exzision empfohlen.^18^, ^19^

In einer prospektiven Studie mit 114 Patientinnen mit VLP entwickelte eine Patientin im Verlauf ein vulväres SCC.^20^ Eine Kohortenstudie zeigte ebenfalls ein erhöhtes Risiko für Vulvakarzinome bei Patientinnen mit VLP.^21^ Die bislang größte Fallserie von 38 LRM‐assoziierten vulvären SCCs wies bei Diagnosestellung eine hohe Rate an inguinalen Metastasen (42 %), an Rezidiven des Vulvakarzinoms in der erkrankten Mukosa (39 %) sowie eine hohe krankheitsbedingte Mortalität (37 %) auf.^22^ Ein derart fulminanter und aggressiver Verlauf zeigte sich auch bei unseren beiden Patientinnen, die trotz kurzer Krankheitsdauer invasive Vulvakarzinome entwickelten: bei Patientin 1 traten Metastasen bereits drei Monate nach Diagnosestellung der dVIN auf, bei Patientin 2 waren Metastasen bereits zum Zeitpunkt der Erstdiagnose des Vulvakarzinoms vorhanden. Zu beachten ist, dass bei der Laservaporisation keine histologische Untersuchung erfolgt, sodass retrospektiv nicht sicher beurteilt werden kann, ob das Vulvakarzinom bei Patientin 1 bereits früher bestanden hatte. Im Gegensatz zum Vulvakarzinom auf dem Boden eines LRM, wurde für aus LRM‐Läsionen hervorgehende Oropharynxkarzinome ein möglicherweise weniger aggressiver Verlauf im Vergleich zu Oropharynxkarzinomen bei Nicht‐OLP‐Patienten diskutiert; die Datenlage hierzu ist jedoch begrenzt.^23^

Obwohl in der Literatur bereits über Patienten mit LRM und daraus resultierendem SCC berichtet wurde, möchten wir auf das Potenzial fulminanter, hochdynamischer und metastasierender Verläufe mit letalem Ausgang aufmerksam machen, insbesondere beim VLP. Daher sollten Patienten mit LRM interdisziplinär betreut werden und regelmäßige dermatologische sowie gynäkologische Kontrolluntersuchungen erhalten. Diese umfassen dermatologische und gynäkologische Untersuchungen, bei denen Haut, orale Mukosa, Genitalregion einschließlich Vulva und Vagina, Analregion, Nägel und Haare systematisch beurteilt werden sollten. Therapieresistente Läsionen sollten regelmäßig biopsiert werden, um eine maligne Transformation auszuschließen. Hinsichtlich spezifischer Untersuchungsintervalle besteht die beste Evidenz für den OLP mit Kontrollen alle 3–4 Monate.^15^ Die Intervalle für gynäkologische Kontrolluntersuchungen sind in der Literatur nicht einheitlich festgelegt. Nach unserer Einschätzung erscheint jedoch auch hier ein Kontrollintervall von 3–4 Monaten sinnvoll, insbesondere vor dem Hintergrund des fulminanten Verlaufs im Falle der malignen Entartung. Eine effektive Therapie des LRM ist erforderlich, um eine potenziell letale maligne Transformation der Läsionen zu verhindern  .

**TABELLE 1:** Patientencharakteristika

	Patientin 1	Patientin 2	Patientin 3
**Geschlecht**	Weiblich	Weiblich	Weiblich
**Alter**	63 Jahre	64 Jahre	77 Jahre
***Body‐Mass‐Index***	28,7	29,9	25,8
**Raucherstatus**	Nie‐Raucher	Nie‐Raucher	Nie‐Raucher, kein Alkoholkonsum
**Komorbidität**	Arterielle Hypertonie, Depression	Arterielle Hypertonie, Hypothyreose, Thrombozytopathie, Depression	Diabetes mellitus Typ 2, arterielle Hypertonie, Osteoporose, Kolonkarzinom (2010)
**Alter bei Erstmanifestation des LRM**	58 Jahre	61 Jahre	44 Jahre
**Alter bei Diagnosestellung des LRM**	60 Jahre	61 Jahre	44 Jahre
**Dauer bis zur Diagnosestellung**	26 Monate	4 Monate	n. a.
**Betroffene Schleimhäute**	Genital und oral	Genital	Oral und genital
**Extramukosale Manifestation**	Nein	Nein	Ja (Haut)
**Familienanamnese bzgl. LRM**	Negativ	Negativ	Positiv (Tochter)
**Bisherige Systemtherapien**	Intravenöse Dexamethason‐Pulstherapie Methotrexat Hydroxychloroquin	Acitretin	Orales Prednisolon Acitretin
**Letzte Therapie vor Diagnosestellung von Malignität**	Topische Therapie	Acitretin	Topische Therapie
**Intervall zwischen Erstmanifestation von LRM und Malignität**	2 Jahre (dVIN)	2 Jahre (Vulvakarzinom)	32 Jahre (Oropharynxkarzinom)
**Intervall zwischen Diagnosestellung von Malignität und Auftreten von Metastasen**	3 Monate	0 Monate	0 Monate
**Therapie nach Diagnosestellung des metastasierten SCC**	Partielle Resektion der Vulva und der vorderen Vaginalwand, Urethraresektion, bilaterale inguinale Lymphknotendissektion Bilaterale laparoskopische pelvine Lymphadenektomie, bilaterale Adnexektomie Radiochemotherapie mit Cisplatin	Bilaterale laparoskopische inguinale Lymphadenektomie Chemotherapie mit Cisplatin und Paclitaxel Vulvektomie mit bilateraler inguinaler Lymphadenektomie Radiotherapie	Laserresektion und bilaterale *Neck Dissection* Linke submandibuläre Lymphadenektomie Radiochemotherapie mit Cisplatin
**Krankheitsverlauf**	2,5 Monate nach Abschluss der Radiochemotherapie fulminante Progression mit kutanen Metastasen, axillären Lymphknotenmetastasen und Peritonealkarzinose	Fünf Monate nach Abschluss der Radiotherapie ausgedehnte Peritonealkarzinose	13 Monate später Wiedervorstellung mit einem nicht resektablen, ausgedehnten lokalen Rezidiv
**Verstorben aufgrund von LRM, Alter**	Ja, 63 Jahre	Ja, 64 Jahre	Ja, 77 Jahre

*Abk*.: dVIN, differenzierte vulväre intraepitheliale Neoplasie; LRM, Lichen ruber mucosae; n. a., nicht anwendbar; SCC, Plattenepithelkarzinom

## DANKSAGUNG

Open access funding enabled and organized by Projekt DEAL.

## INTERESSENKONFLIKT

J. Gallwas war Beraterin und/oder erhielt Vortragshonorare oder nahm an klinischen Studien der folgenden Unternehmen teil: MSD SHARP & DOHME und Novartis Pharma.

R. Mössner war Beraterin und/oder erhielt Vortragshonorare und/oder Zuschüsse und/oder nahm an klinischen Studien der folgenden Unternehmen teil: Abbvie, Almirall, Biogen IDEC, Böhringer‐Ingelheim, Celgene, Janssen‐Cilag, Leo Pharma, Lilly, Moonlake, MSD SHARP & DOHME, Novartis Pharma, Pfizer und UCB.

M. P. Schön war Berater und/oder erhielt Vortragshonorare und/oder Zuschüsse und/oder nahm an klinischen Studien der folgenden Unternehmen teil: AbbVie, Allmirall, Biogen, Böhringer‐Ingelheim, BMS, Celltrion, Janssen‐Cilag, Leo, Lilly, Novartis, Scinai, UCB.

J. Wenzel hat finanzielle Unterstützung (klinische Studien, Reisestipendien, Vortragshonorare) von GlaxoSmithKline, Novartis, Medac, Merck/Serono, Lilly, Bristol Myers Squibb, Roche, Actelion, Pfizer, Spirig, ArrayBio, Biogen und Incyte erhalten.

D. Wilsmann‐Theis war als Beraterin tätig und/oder erhielt Vortragshonorare oder Reisekostenerstattungen und/oder Zuschüsse und/oder nahm an klinischen Studien der folgenden Unternehmen teil: AbbVie, Almirall‐Hermal, Amgen, Biogen, Bristol Myers Squibb, Boehringer Ingelheim, Celgene, Forward Pharma, GlaxoSmithKline, Incyte, Janssen‐Cilag, Klinge Pharma, Leo, Eli Lilly, Medac, Merck, Moonlake, Novartis, Pfizer und UCB.

N. Cramer gibt keine Interessenkonflikte an.
